# Research on Deterioration Mechanism and High-Precision Modelling of the Core Loss for Amorphous Alloys after Wire-Cut Electric Discharge Machining

**DOI:** 10.3390/ma16062275

**Published:** 2023-03-12

**Authors:** Xinyu Yang, Shuheng Qiu, Yuheng Wang, Pengfei Zhao, Yunpeng Gao, Haifeng Wang, Chi Zhang

**Affiliations:** 1 School of Rare Earths, University of Science and Technology of China, Hefei 230026, China; 2 Ganjiang Innovation Academy, Chinese Academy of Sciences, Ganzhou 341119, China; 3 Ningbo Institute of Materials Technology and Engineering, Chinese Academy of Sciences, Ningbo 315201, China; 4 Faculty of Electrical Engineering and Computer Science, Ningbo University, Ningbo 315211, China

**Keywords:** amorphous alloy, wire-cut electric discharge machining, performance deterioration mechanism, domain distribution quantification, affected area division, modified core loss model

## Abstract

Amorphous alloys (AAs) have the advantage of low core loss. Thus, they can be used in high-speed motor applications. However, compared with the nominal performances, the performance of the wire-cut electric discharge machine (W-EDM)-processed AA iron core changes significantly, which limits its popularization. This paper focuses on the performance degradation mechanism of the AA ribbon caused by W-EDM and establishes a modified core loss model after machining. First, a 308 × 15 mm ribbon-shaped AA sample machined by W-EDM was prepared. The characterization and analysis of the magnetic properties, phase, magnetic domain, nano-indentation, micro-morphology, and composition were carried out. In this paper, by analysing the variation in the magnetic domain distribution based on domain width and nano-mechanical properties, it is proposed that the performance degradation range of AA ribbons processed by W-EDM is within 1 mm from the edge. By comparing the microscopic morphology and chemical composition changes in the affected and the unaffected area, this paper presents a mechanism for the property deterioration of W-EDM-processed AA ribbons based on electrochemical corrosion. Finally, a modified loss model for W-EDM-processed AAs is established based on the division of the affected area. This model can significantly improve the accuracy of core loss estimation in the medium- and high-frequency bands commonly used in high-speed motors.

## 1. Introduction

Amorphous alloys (AAs) have the advantage of low core loss [1,2], which can replace traditional silicon steel sheets in high-frequency transformers and high-speed motor cores designed for high power density applications [3,4]. The process of manufacturing an iron core from AA ribbons is in the order of fragmentation, lamination, annealing, clamping, curing and cutting. Similar to silicon steel sheets [5], the loss properties of AA ribbons are degraded after being cut into cores of the expected shape, but the decrease is much greater than that of other soft magnetic materials [6,7]. In comparison, Chai et al. [8] concluded that the slot opening of the amorphous alloy core has a significant effect on the core loss, whose maximum increase can reach 30%. Due to the performance degradation, the actual performance of the AA-based electrical machines has a large discrepancy between the simulation results, which increases the difficulty and cost during the design stage [9]. Therefore, establishing the performance degradation model can effectively improve the accuracy and speed in electrical machine design and styling.

The cutting methods for AA cores mainly include punching [10], laser-cutting [11], water-jet [12], and wire-cut electrical discharge machining (W-EDM) [13]. Celie et al. [10] studied the performance of a high-speed motor formed by punched and laser-cut AA ribbons. The results showed that annealing can effectively improve the deterioration of loss characteristics caused by punching. However, they also proposed that the effect of laser cutting on performance is irreversible. Liu et al. [13] compared the ultra-high-speed motor rotors processed by laser cutting and W-EDM. Furthermore, it was found that the edge of the laser-cut core is ablated, resulting in high eddy current loss induced by inter-layer short circuits, while the wire-cut iron core had better edge quality. Qu et al. [14] also compared the cutting quality and magnetic properties of punching, laser-cutting, and W-EDM on AA cores and ribbons, respectively. Furthermore, the conclusion stated that W-EDM-processed AA cored had the best comprehensive performance. Among the above processing methods, W-EDM realizes material reduction manufacturing through non-electrostatic discharge between the moving wire tool electrode (molybdenum wire) and the workpiece [15], which is most suitable for forming AA cores due to the cutting thickness, precision, and cost advantages [16]. The research on AA applications mainly focuses on the macro electromagnetic performance of the processed ribbons or cores. The loss simulation is corrected by constant coefficients or look-up tables. However, the mechanism of performance degradation is still lacking.

Microscopic observation of the microstructure changes of AAs can explain the mechanism of performance degradation caused by processing. Yan et al. [17] used SEM to observe the fracture surface of roll-sheared AA ribbons. The results show that the shear section of Fe-based AA ribbons mainly had the characteristics of a collapse angle, fracture slip rub zone and burrd. Katakam et al. [18] established a thermal model to predicate the temperature of the edge area of laser-cut AA ribbons would be higher than the crystallization temperature. They found crystallization occurred in the cutting area by photographing the bright-field TEM micrographs, which verified the prediction [19]. Szabo et al. [20] found that there is a heat-affected zone (HAZ) in laser-cut amorphous alloy ribbons, the size of which is positively related to laser intensity. The HAZ consists predominantly of the crystallized part of the ribbon in the vicinity and parallel to the cutting front, where hardness increases [21]. The SEM figure shows a columnar microstructure within the HAZ, resembling a typical re-solidified microstructure. Furthermore, the columnar increasingly finer dispersity in the deeper region of HAZ [22]. The size of the HAZ region observed microscopically has good correspondence with micro-hardness, coercivity, and saturation magnetization [23]. Guo et al. [24] studied the effects of pulsed-laser processing (PLP) on the surface morphology, structural transformation, and crystallization kinetics of a Fe${}_{78}$Si${}_{9}$B${}_{13}$ amorphous alloy. They divided the AA ribbon, processed by PLP, into three regions, namely, the melting zone, the heat-affected zone, and the unaffected zone, with different mechanical and magnetic properties [25]. In addition, magnetic domain observations are also a common method to characterize the magnetic properties of ribbons. Senda et al. [26] found that the edge of sheared silicon steel strips exhibit three magnetic domain regions with different structures. Meanwhile, the area of core loss degradation was estimated to be 1.5–2.5 mm. The study of micro-morphology, qualitatively analysed the mechanism of performance deterioration of processed AA ribbons, but there is still a lack of correspondence with actual physical properties.

The performance of AA ribbons decreases significantly after being processed into electrical machine cores. In this paper, the mechanism and core loss modelling of performance degradation of AA ribbons induced by W-EDM are studied. Firstly, we obtained the changes in the samples after W-EDM, including the cross-sectional morphology, magnetic properties, and phase changes. Furthermore, magneto-optical Kerr microscopy was used to observe the magnetic domain structure within 3340 μm of the edge of the W-EDM AA ribbon. By counting the proportion of magnetic domains with different widths at different positions, the variation rule of their widths with the distance from the cutting edge was obtained, by which the influence area of wire cutting was divided to 1 mm. The hardness change of the sample was also tested. Next, the microstructure and chemical composition analysis of the different influence areas were characterized to explore and explain the reasons for the above changes. The mechanism of performance degradation was oxidation and corrosion of the surface. Finally, this paper proposes a modified calculation method of core loss based on influence partition, in which $R^{2}$ reaches 0.9994 at different frequencies. The relative error reaches 1.6% at 400 Hz.

The following parts of this paper are arranged as follows. In Section 2, the material, preparation method of AA ribbons, and the characterization instrument is introduced. The changes in properties, magnetic domain structure, and the division of the influence area of W-EDM are presented in Section 3. Section 4 provides the characterization results by micromorphology and component analysis, which clarifies the mechanism of performance change. In Section 5, the modified core loss model for W-EDM-processed AA ribbons is presented. Finally, the conclusion is presented in Section 6.

## 2. Experimental Samples and Instruments

### 2.1. Sample Preparation

Iron-based AA ribbons (Antai Technology & Materials Co., Ltd., Beijing, China. Brand: 1K101) with a nominal composition of Fe${}_{80}$Si${}_{9}$B${}_{11}$ in atomic percentage and a thickness of 24 μm are used for the W-EDM cutting experiments. The basic information and nominal performance of the material are given in Table 1.

**Figure 1:**
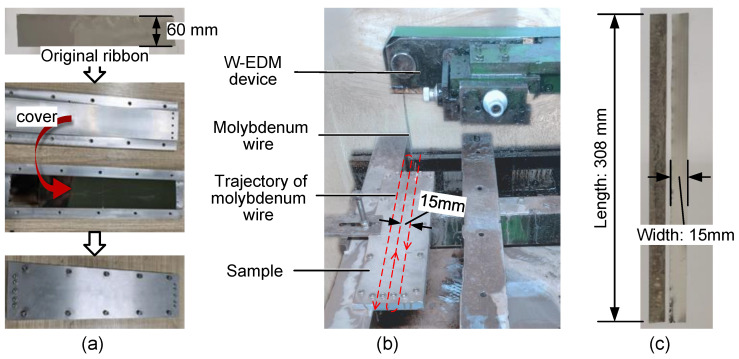
Sample preparation process. (**a**) Original ribbon and aluminium tooling, (**b**) W-EDM equipment and trajectory of molybdenum wire. (**c**) Prepared AA samples.

The ribbons were cut into samples with 308 × 15 mm dimensions for the experiments. In the process of sample preparation, the aluminium tooling shown in Figure 1a was used to clamp a single ribbon for W-EDM cutting to overcome the problem of the ribbons being too thin. The W-EDM equipment and trajectory of the molybdenum wire are shown in Figure 1b. The pulse EDM width was 50 μs, and the current was 2 A. Finally, The samples were annealed at 390 °C for 96 min to remove residual stresses caused by machining. The prepared samples are shown in Figure 1c.

### 2.2. Magnetic Measurements

**Figure 2:**
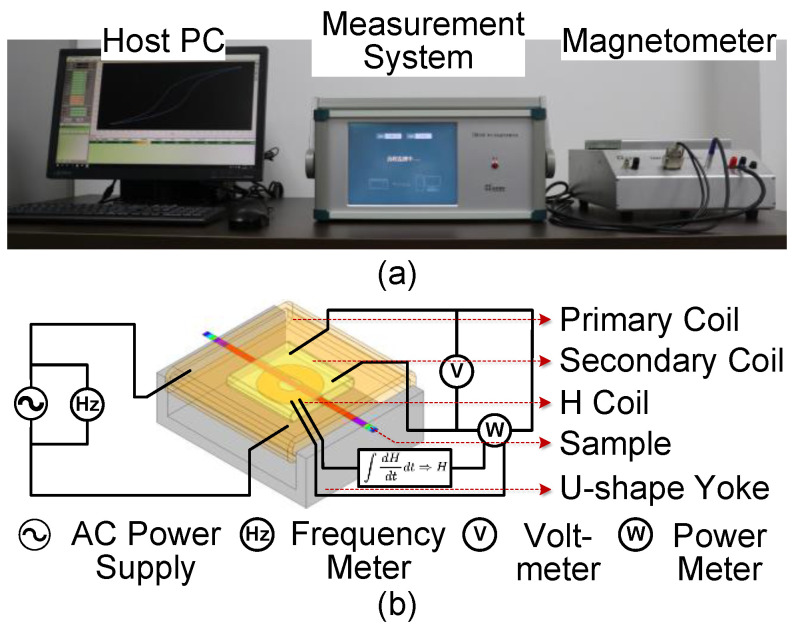
TD8160 monolithic AA magnetic tester system. (**a**) Actual measuring device, (**b**) Basic structure of magnetometer and measurement system.

The monolithic AA magnetic tester system, TD8160(TUNKIA, Changsha, China), with ±2% nominal error, was used for magnetic measurement of the prepared samples, which is shown in Figure 2a. The magnetometer consists of a U-shape high magnetic permeability yoke, primary coil, secondary coil and H-coil [27]. The composition of each component of the magnetometer and its connection with the measurement system is shown in Figure 2b. Furthermore, the prepared AA samples were inserted between two coils to complete the magnetic test. Core losses of the AA samples and original ribbons were evaluated under sinusoidal wave forms with frequency of 50, 100, 200, and 400 Hz and magnetic flux densities from 0.1 to 1.5 T.

### 2.3. Phase and Morphological Characterization

Due to the serious stains on the surface of the sample processed by W-EDM, the surface quality of the sample was improved by anhydrous ethanol and 500 s of plasma polishing successively before phase and morphology characterization. The material characterization methods used in this paper include phase analysis, mechanical properties analysis, magnetic domain analysis, surface morphology analysis, and chemical composition analysis.

The X-ray diffraction (XRD, German Bruker, Karlsruhe, Germany) patterns of crystalline materials and amorphous materials show sharp and diffuse diffraction peaks, respectively. Thus, XRD can be used for phase analysis of different materials. Here, the phase analysis for diffraction angle (2θ) of 20∼80${}^{\circ }$ is based on German Bruker D-8 XRD analyzer with Cu-Kα radiation (λ = 0.154056 nm). Transmission electron microscopy (TEM, FEI, Hillsboro, OR, USA, Tecnai F20) was used to further observe the microstructure of the samples. The amorphous ribbon were cut into 3 mm discs, and then an ion thinner was used until a hole formed. The data obtained were processed using the Digital Micrograph software.

**Figure 3:**
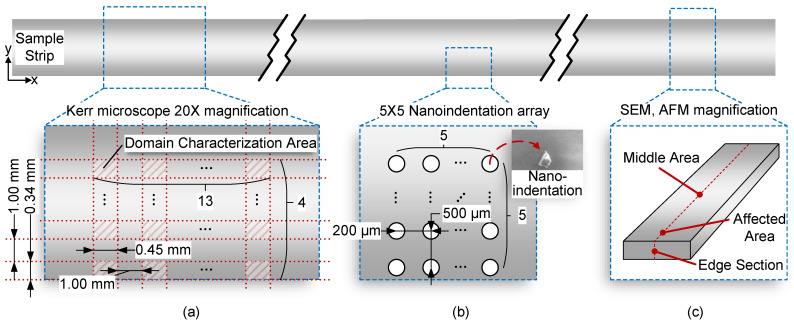
Micro characterization scheme. (**a**) Observation scheme for the magneto-optical Kerr microscope. (**b**) Nano-indentation characterization scheme. (**c**) Micro characterization scheme.

The Kerr effect refers to the phenomenon when the polarization plane deflects plane polarized light reflects on the surface of a magnetic material [28], which can be used to observe the magnetic domain structure on the surface of the samples [29]. Therefore, a magneto-optical Kerr microscope (Evico magnetics GmbH, Dresden, Germany) with 20× objective lens was used to characterize domain images at room temperature. The scheme of magnetic domain characterization is shown in Figure 3a. The size of the magnetic domain area that can be photographed by the microscope is 0.45 mm × 0.34 mm. From the edge of W-EDM processing, one characterization point was taken every 1 mm between the horizontal and vertical directions. In the area to be characterized, a totally 52 (13 × 4 lattices) magnetic domain figures were taken for further statistical analysis.

The mechanical test adopted the nano-indentation method with constant maximum displacement. By pressing the indenter into the sample according to the predetermined loading curve, when the set maximum displacement is reached, the indenter will unload again in a controlled manner. The required load during loading and unloading was recorded, and the curve between the force and displacement of pressing was finally obtained. Further, the parameters such as hardness and elastic modulus can be obtained from the maximum load, the shape of the indenter and the depth of the indentation. The samples were measured with a maximum displacement of 1000 nm and a holding time of 1 s by the Berkovich nano-indenter (TI750 TriboIndenter, Hysitron Corporation, USA). The indentation point is shown in Figure 3b. There were 25 (5 × 5) points in the nano-indentation lattice. The starting point of the lattice was on the edge of the processed sample. The distance between two longitudinal points was 500 μm, and the distance between two transverse points was 200 μm.

Finally, microscopic characterization by scanning electron microscopy (SEM, SUPRA55, Zeiss, Oberkochen, Germany) was used to observe the surface morphology of the samples, as shown in Figure 3c. The processing edge section, affected area and middle area of the samples were characterized, respectively, by surface morphology. In addition, combined with SEM and electronic dispersion spectrometer (EDS) with an accelerating voltage of 20 kV, the chemical compositions and distributions at different locations were qualitatively analysed, respectively. Raman spectroscopy was used to study the composition of the surface material of the samples. Raman spectra were obtained with the help of a confocal micro Raman spectrometer (In Via Raman Microscope, Renishaw, London, UK) equipped with a 532 nm wavelength detector with the power set was 1%. A spectral range of 100 to 1400 cm${}^{-1}$ Raman shift was scanned for the different areas of the sample. Each sample was scanned at different locations to ensure the reliability of the results. X-ray photoelectron spectroscopy (XPS) is one of the important surface analysis technologies. The XPS spectrum was obtained using an AXIS ULTRA DLD analyser produced by the Shimadzu Company of Japan with Al-Kα radiation, and the XPS data obtained were analysed by the CasaXPS software for peak fitting. The surface roughness of the samples was characterized by a scanning probe microscope (SPM, Dimension 3100, Vecco, New York, NY, USA). The size of the area was 10 μm × 10 μm. The standard roughness estimation parameter $R_{a}$ was used to describe the surface roughness.

## 3. Results and Discussion

### 3.1. Sample Changes after W-EDM Processing

**Figure 4:**
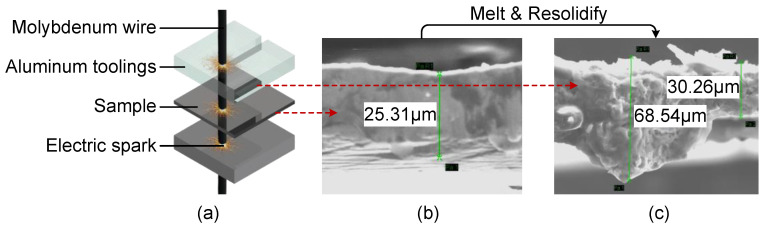
Section morphology results of samples before and after W-EDM. (**a**) Schematic diagram of sample in W-EDM. (**b**) Section morphology of the original ribbon. (**c**) Section morphology of the sample after W-EDM.

W-EDM is a processing method that uses the pulse discharge between a molybdenum wire and the workpiece to generate high-heat to melt or vaporize the workpiece material, the cutting processing of which is shown in Figure 4a. The edge view of the original ribbon is shown in Figure 4b, where the ribbon thickness is consistent with the nominal value of the material with a uniform distribution. However, the sample edge after W-EDM processing shows the characteristics of thickening and obvious undulation, shown in Figure 4c. Therefore, it can be considered that the edge of the AA sample melted and re-solidified under the action of high temperature caused by the electric spark of W-EDM.

In addition to the morphology changes on the processed edge, the magnetic properties of AA samples also deteriorated significantly after W-EDM processing.

**Figure 5:**
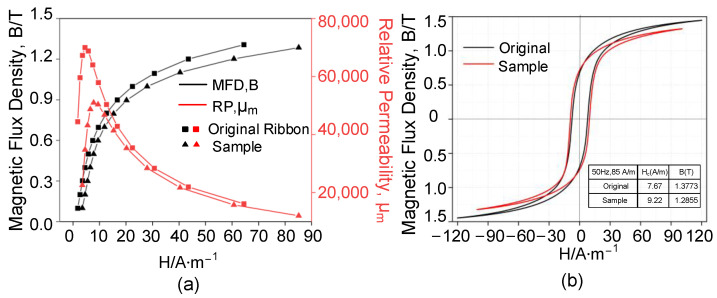
Magnetic test results of the original ribbon and processed sample. (**a**) Magnetization curve and magnetic permeability curve. (**b**) The magnetic hysteresis loops at 50Hz.

The primary magnetization curves and magnetic hysteresis loops of the W-EDM-prepared sample and original ribbon are shown in Figure 5. As shown in Figure 5a, compared with the original ribbon, the saturation magnetic density of the AA samples after processing slightly decreased. Furthermore, the corresponding dynamic magnetic hysteresis loops are shown in Figure 5b. At 50 Hz and 85 A/m magnetic field intensity, the magnetic induction intensity of the original ribbon was about 1.38 T, similar to the nominal properties in Table 1. However, the sample processed by W-EDM was about 1.29 T, indicating the decline in magnetic properties. Furthermore, under the same conditions, the corresponding $H_{c}$ of the sample after W-EDM was higher. Due to the thin sample, the eddy current loss was relatively small, so the $H_{c}$ is an important parameter to determine the loss.

Furthermore, its magnetic permeability also significantly deteriorated. As shown in Figure 5a, the peak relative permeability of the W-EDM-processed sample was about 72.8% of the original ribbon. According to Equation (1) [30], under the conditions of the same peak magnetic flux density, the decrease in the magnetic permeability leads to an increase in the core loss of the material.

(1) $$P_{iron}=\frac{2\pi^{2}\tau }{\mu }B^{2}f$$

where $P_{iron}$ represents the loss per cycle under a sinusoidal field, τ represents the time constant of the delayed response to time changes of the field, μ represents the magnetic permeability, *B* represents the magnetic flux density peak value in the cycle, and *f* represents the frequency of a sinusoidal field.

**Figure 6:**
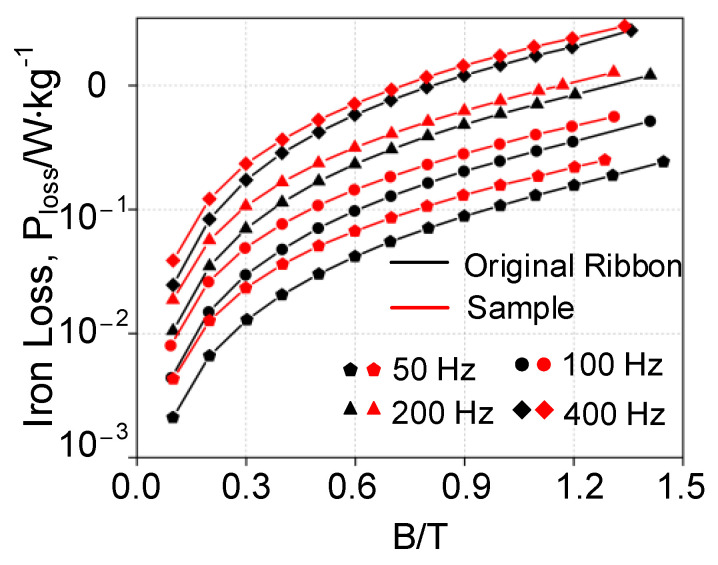
Core loss curves of the AA samples at different frequencies.

The core loss of the AA samples at different frequencies is shown in Figure 6, in which the loss of the W-EDM-processed sample was significantly higher than the original ribbon. Taking the condition of 1.2 T and 400 Hz as an example, the W-EDM process increases the core loss of the ribbon by around 1.2 times.

### 3.2. Phase Analysis

Due to the poor thermal stability of AAs, the transient high temperature caused by the W-EDM spark may induce nanocrystalline precipitation. Thus, XRD and TEM were used to analyse the phase of the processed samples.

**Figure 7:**
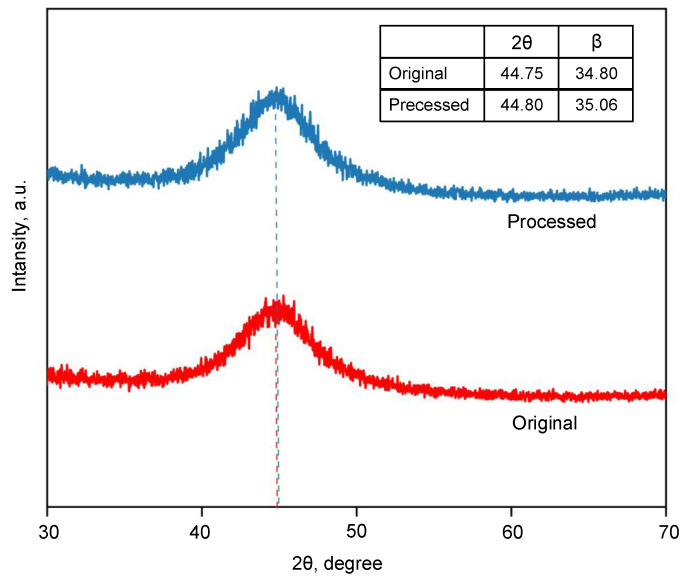
XRD patterns of the original and processed AA ribbons.

The XRD patterns of the original and processed AA ribbons are shown in Figure 7. The XRD pattern of the samples before and after W-EDM all show a diffuse diffraction peak at $2\theta \approx 44.8^{\circ }$. The values of the full width at half maximum (β) have little differences. The results show that the W-EDM-processed sample has no obvious crystallization behaviour and still had a high content of the amorphous phase.

**Figure 8:**
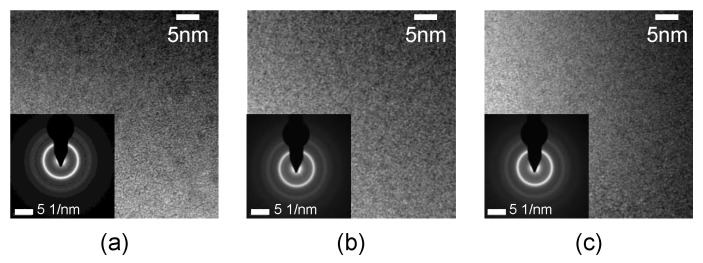
TEM images and selected area diffraction patterns of AA samples. (**a**) HRTEM and SAED of the original ribbon, (**b**) HRTEM and SAED of the centre on the processed sample. (**c**) HRTEM and SAED of the cut edge on the processed sample.

Figure 8 shows the TEM results of the AA ribbon before and after W-EDM processing, which includes the high-resolution transmission electron microscopy (HRTEM) images and selected area electron diffraction (SAED) patterns in the lower-left corner. The original ribbon, centre area, and edge area of the processed sample were characterized by TEM separately, as shown in Figure 8a–c. The HRTEM images of the three all show an almost completely disordered structure. Furthermore, the corresponding SAED patterns also show diffuse diffraction rings. The above phenomena all meet the typical amorphous structure.

Combined with the results of XRD and TEM, it can be concluded that the edge and centre of the AA samples after W-EDM processing still maintain the same disordered structure as the original ribbon. This shows that there is no crystal or nano-crystal precipitation in the samples after W-EDM processing.

### 3.3. Magnetic Domain Analysis

The broad and regular domain structure indicates low domain energy and good soft magnetic properties [31], while the finely twisted domain structure hinders wall motion during magnetization, leading to increased hysteresis losses [29].

**Figure 9:**
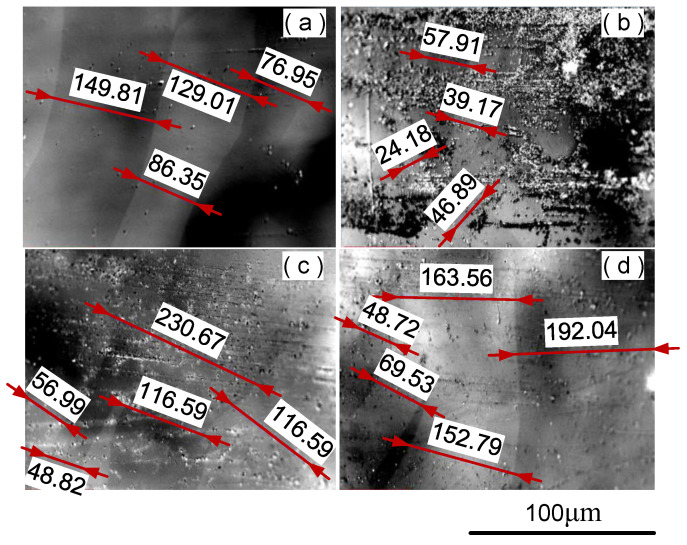
Magnetic domain image of samples before and after W-EDM processing. (**a**) Original ribbon, (**b**) 0 mm, (**c**) 1 mm, and (**d**) 3 mm from the cut edge.

According to the domain characterization scheme proposed in Section 2.3, a total of 52 magnetic domain images were photographed. The characteristics of typical magnetic domains at different horizontal positions are shown in Figure 9. The magnetic domain of the original ribbon is shown in Figure 9a, which presents a wide and regular stripe pattern. It shows that the original ribbon has a uniform structure, low-stress state, and good soft magnetic properties. After W-EDM processing, the samples have irregular domain shapes. Figure 9b–d show typical domain patterns at 0, 1, and 3 mm from the edge, respectively. It can be seen that the magnetic domain width at the edge of the processed sample is narrow, and the number of domain walls increases significantly. However, at 3 mm from the cutting edge, a wide and regular magnetic domain state similar to that of the original ribbon appears again. It was found that the proportion of wide magnetic domains increases with the increasing distance from the edge of W-EDM processing.

**Figure 10:**
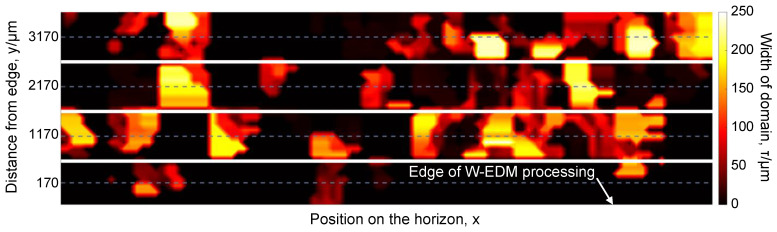
Distribution of different width magnetic domains after W-EDM processing.

By counting the width in the above magnetic domain images, the domain width distribution contour can be plotted as shown in Figure 10. It also shows that the closer the magnetic domain is to the edge, the more affected it is, especially in a distance within 1 mm.

### 3.4. Nanoscale Mechanical Property

**Figure 11:**
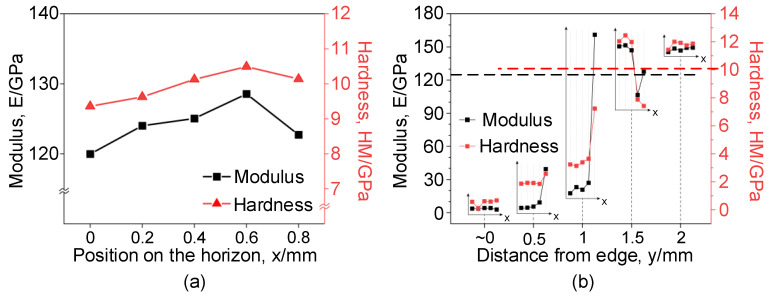
Nanoscale mechanical properties of samples at different positions, including elastic modulus and hardness, before and after W-EDM. (**a**) Nanoscale mechanical properties of the original ribbon, and (**b**) processed samples. The black dotted line represents the modulus of the original ribbon, and the red dotted line represents the hardness of the original ribbon.

The re-solidification at the edge of the W-EDM-processed sample leads to changes in the mechanical properties of the metal material. The elastic modulus and hardness of the samples at different positions can be measured by nano-indentation, as shown in Figure 11.

The nanoscale mechanical properties of the original ribbon are shown in Figure 11a, with the elastic modulus and hardness around 130 GPa and 9 GPa, respectively. The nanoscale mechanical properties at different positions on the sample after W-EDM processing are shown in Figure 11b. Nano-indentation on the same horizontal line (x direction) basically shows consistent performance. The elastic modulus and hardness closer to the processed edge are significantly lower than the original ribbon, decreasing by 80.7 and 91.4%, respectively, in the region close to the processed edge. However, at the centre of the processed sample, the elastic model and hardness are still relatively close to the original ribbon.

### 3.5. Affected Areas on the W-EDM-Processed AA Ribbon

The above experimental results show that the domain width distribution contour, in Figure 10, hardness and elastic modulus, in Figure 11, all show regular changes with the distance from the W-EDM-processed edge. The region within 1 mm from the W-EDM-processed edge exhibits the properties of narrow domain width, low hardness, and modulus. However, the area beyond 1 mm from the processed edge, all parameters are close to those of the original ribbon.

Therefore, the influence area of the W-EDM process on AAs can be determined as the range within 1 mm from the processed edge. In this region, the magnetic properties and mechanical properties of the AA ribbon have significantly deteriorated.

## 4. Mechanism

### 4.1. Morphology and Composition

**Figure 12:**
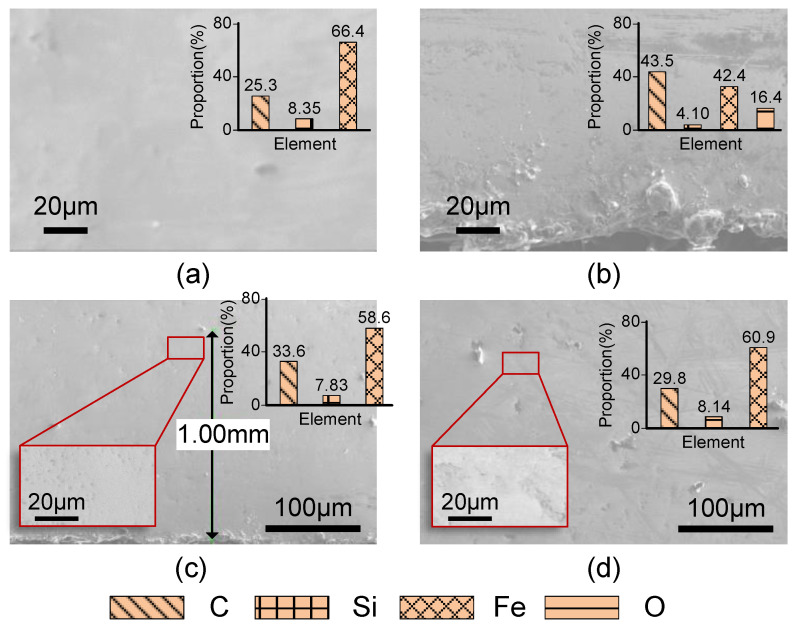
SEM and EDS results of AA ribbons. (**a**) Original ribbon; (**b**) edge of W-EDM-processed sample; (**c**) affected area of the processed sample; (**d**) unaffected area of the processed sample.

Under SEM, the surface morphology of different regions on the sample processed by W-EDM can be observed. The surface morphology of the original ribbon is shown as Figure 12a, which is smooth and flat. The edge of the W-EDM-processed sample is shown in Figure 12b. There are obvious processing marks on the surface. Re-solidification on the edges causes surface roughness and produces burrs. In the 1 mm area that is affected by W-EDM processing, the microscopic morphology is shown in Figure 12c. Some small dimples and protrusions are still visible. However, at the centre of the processed sample, its smooth and flat state is consistent with the original ribbon, as shown in Figure 12d.

It is obvious that the affected area on the processed sample becomes uneven. This results in the composite interface formed between the bottom surface of the droplet and the groove, more easily transitioning from the unstable Cassie state to the Wenzel state when the cooling liquid droplet comes in contact with the sample surface. This leads to increased wettability of the metal surface [32]. The cooling liquid is more likely to remain on the edge of the sample after W-EDM processing, forming a water film. Under the joint action of water and air, the surface of the sample edge is more prone to oxidation and corrosion. Therefore, under EDS analysis, as shown in Figure 12b, a higher proportion of oxygen atoms appears in the affected area.

Furthermore, the EDS results of the original ribbons are shown in the insets in the upper-right corner of Figure 12d. It is shown that the surface contains about 25.3 at.% C, 8.35 at.% Si, and 66.35 at.% Fe. After W-EDM processing, the elemental composition in the affected area near the edge changed greatly. However, as the distance from the edge increases, the composition of the unaffected area converges to that of the original ribbon.

**Figure 13:**
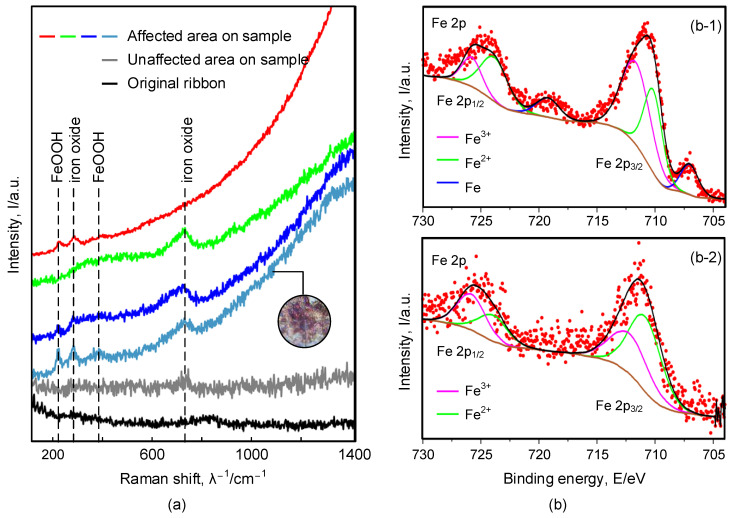
(**a**) Raman spectra of Fe${}_{80}$Si${}_{9}$B${}_{13}$ samples before and after W-EDM, showing characteristic peaks of iron oxide and oxyhydroxide phases. (**b**) XPS spectra of Fe${}_{80}$Si${}_{9}$B${}_{13}$ ribbons before W-EDM (**b-1**) and after W-EDM (**b-2**), showing characteristic peaks of iron oxide and oxyhydroxide phases.

The composition of the processed samples characterized by Raman spectroscopy is shown in Figure 13a. The spectrum shows that iron (III) oxide and iron hydroxide oxide exist on the surface of the sample in the affected area caused by W-EDM processing. Likewise, the XPS spectra also confirm the changes in the composition on the processed sample surface, as shown in Figure 13b. Figure 13(b-1) is the XPS spectrum of the original ribbon, showing the existence of iron (binding energies of 707.1 eV). Figure 13(b-2) shows the state of iron at the edge of the W-EDM-processed sample. The binding energies of Fe${}^{2+}$ and Fe${}^{3+}$ in the 2p 3/2 and 2p 1/2 energy levels are 710.8, 712.2 eV and 724.0, 725.0 eV, respectively, [33], which are all reflected in the XPS spectrum.

**Figure 14:**
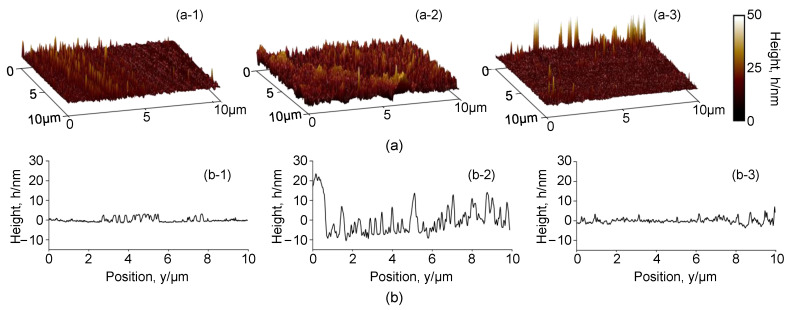
Surface profile of AA samples before and after W-EDM processing: (**a**) 3D profile of AA samples before and after W-EDM processing. (**a-1**) the original ribbon, (**a-2**) the W-EDM-processed edge, and (**a-3**) the unaffected area on the processed sample. (**b**) Surface height distribution of AA samples before and after W-EDM processing. (**b-1**) the original ribbon, (**b-2**) the W-EDM-processed edge, and (**b-3**) the unaffected area on the processed sample.

The surface profile of the samples before and after W-EDM processing was further observed by AFM, as shown in Figure 14a. Figure 14(a-1–a-3) display the surface profiles of the original ribbon, the W-EDM-processed edge, and the unaffected area on the processed sample, respectively. Their average surface roughness was 2.03, 4.68, and 1.57 nm, respectively. Figure 14b shows the height distribution at different locations, and corresponds to Figure 14a, where the abscissa is the vertical direction (i.e., perpendicular to the W-EDM-processing edge). It is obvious that the height variation on the edge region of the processed sample is more in-homogeneous.

### 4.2. Mechanism of Performance Deterioration

**Figure 15:**
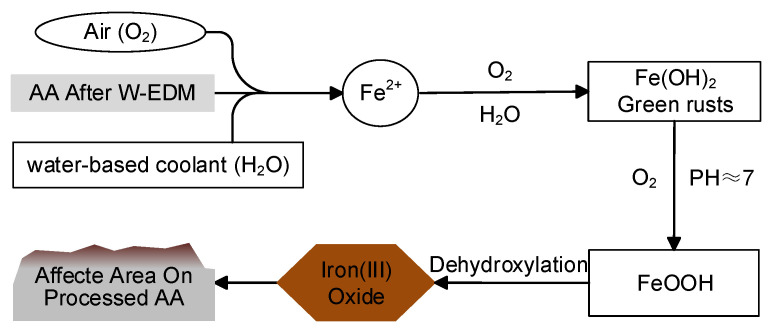
Schematic diagram of the mechanism.

During the W-EDM processing, the water-based coolant is sprayed intermittently onto the workpiece around the molybdenum wire to cool the high temperatures generated by the electric spark. The coolant forms a liquid film at the processing edge of the AA ribbon due to the unevenness caused by the W-EDM erosion. The iron in AA, the water-based coolant, and the oxygen-enriched air environment constitute the galvanic reaction. On the surface of the AA ribbon, Fe${}^{2+}$ ions are continuously precipitated into the liquid film to form Fe(OH)${}_{2}$ and its hydrate. The ferric hydroxide reacts with oxygen in the collet of neutral or weak-alkali to form a precipitate mainly composed of ferric oxyhydroxide. Ferric oxyhydroxide undergoes further dehydration reactions to form iron (III) oxide [34]. However, for the unaffected area on the W-EDM-processed AA ribbon, the surface is relatively smooth, which makes it difficult for the coolant to remain. At the same time, the oxidation protection layer on the original surface is not damaged by erosion. Therefore, electrochemical corrosion is difficult to occur, so the unaffected area basically maintains the same morphology and performance as the original ribbon. The above process is shown in Figure 15.

Due to the spraying of the coolant, the re-solidification of the eroded AA still meets the conditions of rapid quenching. Thus, the TEM result still shows an amorphous state, as shown in Figure 8. The rapid cooling leads to a rapid “freezing” of the internal atoms, increasing the tendency of loose atomic packing and “liquid-like domains”, which reduces the mechanical properties of the affected areas, as shown in Figure 11. Furthermore, the corrosion product is too thin to produce higher XRD diffraction peaks, so it cannot be identified in Figure 7. The iron (III) oxide structure produced on the surface of AA ribbon is loose, resulting in a rough surface, as shown in Figure 14. The iron (III) oxide has the characteristics of high coercive force and iron loss, which leads to the deterioration of loss performance after W-EDM processing. In addition, the formation of oxides makes it difficult to clearly observe the magnetic domain morphology, resulting in the overall domain distribution as shown in Figure 10. Surface oxides also lead to tensile stress in the amorphous matrix. These stresses are the origin of pinning points, restricting the motion of magnetic domains and further degrading the magnetic properties on the processed edge.

## 5. Core Loss-Modified Model for AA Ribbons

### 5.1. Loss Model Establishment

**Figure 16:**
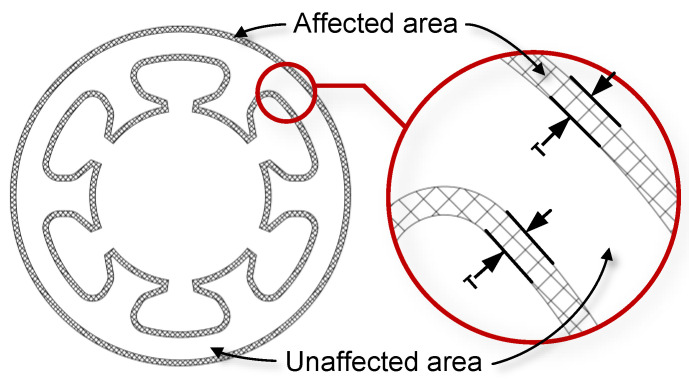
Schematic diagram of the loss model of the sample.

Based on the loss deterioration mechanism of W-EDM-processed AA ribbons, a modified loss model of processed AAs suitable for engineering calculation was established. As shown in Figure 16, the loss calculation area is divided according to the W-EDM-processing-affected area. Therefore, the loss, $P_{loss}$, calculation of the AAs were revised as Equation (2).

(2) $$P_{loss}=V\rho {\hat{P}}_{mod}=2hl\rho \int_{0}^{\tau }\mu_{mod}\;P_{o}\;dx+hl\rho \int_{\tau }^{w-\tau }\;P_{o}\;dx$$

where *V* and ρ represents the volume and density of the processed AA ribbons, respectively, ${\hat{P}}_{mod}$ represents the estimated core loss density, *h* represents the thickness of the ribbon, *l* represents the length of the ribbons, *w* represents the width of the ribbons, τ represents the width of the affected area, $\mu_{mod}$ represents the modified factor, and $P_{o}$ represents the nominal loss density of AA ribbons.

Equation (3) can be carried out by simplifying Equation (2).

(3) $${\hat{P}}_{mod}=\frac{2\tau \mu_{mod}+w-2\tau }{w}P_{o}$$

Substituting the measured and nominal loss of AA ribbons into Equation (3), the over-determined equation shown in Equation (4) can be obtained.

(4) $$\left[\begin{array}{c} {\hat{p}}_{mod_{1}}\\ {\hat{p}}_{mod_{2}}\\ \vdots \\ {\hat{p}}_{mod_{n}} \end{array}\right]=\frac{2\tau }{w}\left[\begin{array}{cccc} \mu_{mod} & 0 & \cdots & 0\\ 0 & \mu_{mod} & \cdots & 0\\ 0 & 0 & \ddots & \vdots \\ 0 & 0 & \cdots & \mu_{mod} \end{array}\right]\left[\begin{array}{c} p_{o_{1}}\\ p_{o_{2}}\\ \vdots \\ p_{o_{n}} \end{array}\right]+\frac{w-2\tau }{w}\left[\begin{array}{c} p_{o_{1}}\\ p_{o_{2}}\\ \vdots \\ p_{o_{n}} \end{array}\right]$$

where ${\hat{p}}_{mod_{1}}$, ${\hat{p}}_{mod_{2}}$, …, ${\hat{p}}_{mod_{n}}$ represent the estimated loss of the W-EDM-processed samples, and $p_{o_{1}}$, $p_{o_{2}}$, …, $p_{o_{n}}$ represent the nominal loss of the original ribbons.

Thus, the least squares solution of the modified factor, $\mu_{mod}$, can be obtained by solving the generalized inverse matrix, as shown in Equation (5).

(5) $$\mu_{mod}=\frac{w}{2\tau }\left({\hat{P}}_{MOD}-\frac{w-2\tau }{w}{\hat{P}}_{O}\right)\cdot {\hat{P}}_{O}^{-1}$$

where ${\hat{P}}_{MOD}$ represent the estimated loss vector, $P_{O}$ represent the nominal loss vector.

**Figure 17:**
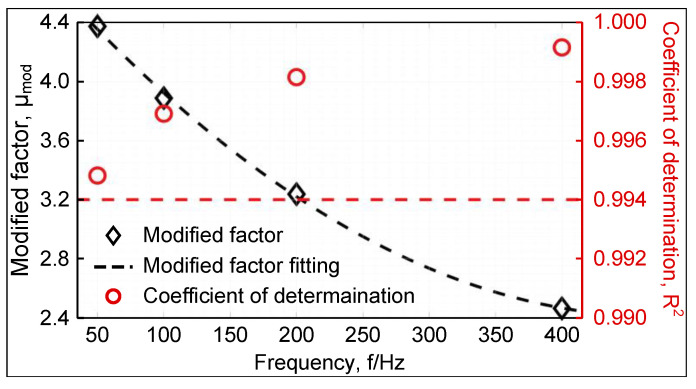
Loss correction factor μ and $R^{2}$ at different frequencies.

The core loss-modified factor with different frequencies can be obtained as shown in Figure 17. The coefficients of determination, $R^{2}$ of the least squares solution are also given in the figure, all of which were higher than 0.994.

The relationship between the modified factor and the frequency can be obtained by fitting the quadratic function to satisfy Equation (6).

(6) $$\mu_{mod}=1.085\times 10^{-5}f^{2}-0.01029f+4.845$$

where *f* represents the frequency.

### 5.2. Loss Model Verification

Finally, the established loss model of W-EDM-processed AAs with the modified factor $\mu_{mod}$ is validated by the data of the validation set measured in the experiment. The results are shown in Table 2.

In the band of industrial frequency (50/60 Hz), the modified model proposed in this paper has little improvement on the accuracy of core loss estimation. However, as the frequency increases, the accuracy of the modified model increases in the mid-to-high-frequency band (≥160Hz) commonly used in high-speed motor applications. Taking a high-speed motor with one pole pair as an example, when the speed is 24,000 rpm, the alternating frequency of the magnetic field is 400 Hz. At this time, the relative error of the modified model is only 1.6%. Therefore, the core loss-modified model proposed in this paper can greatly improve the accuracy of the core loss evaluation of high-speed motors.

## 6. Conclusions

This paper studied the mechanism of deterioration of the magnetic properties of AAs caused by W-EDM processing. Subsequently, a large-scale magnetic domain distribution analysis method based on width analysis was proposed to determine the processing-affected area. Finally, the modified core loss model was presented. The main conclusions are as follows:

1. Large magnetic domains in the area near the edge of the AA ribbon after W-EDM were observed. Furthermore, the characteristic distribution of magnetic domains was obtained based on domain width. Then the variation law of the magnetic domain distribution with the distance from the processed edge was found.
2. Combined with the variation in magnetic domain distribution and nano-mechanical properties with the distance from the processed edge, the affected area of W-EDM processing on the AA ribbon was divided. Furthermore, the range of the affected area was determined to be 1 mm.
3. Through the characterization of surface morphology and chemical composition, the mechanism of magnetic property degradation in the edge region of the W-EDM-processed AA ribbons based on surface corrosion was proposed.
4. A modified model of core loss applied to high-speed motors employing AAs was established. In the mid-to-high-frequency bands, commonly used in high-speed motor applications, the minimum relative error was reduced to 1.6%.

To sum up, this paper proposes a magnetic domain quantitative analysis method; clarifies the affected area of W-EDM-processed AAs; demonstrates the mechanism of magnetic property deterioration on W-EDM-processed AAs; and establishes a high-precision-modified core loss model. The above conclusions are helpful to the high-efficiency design and rational processing of high-speed motors, which are of great significance to the further energy saving of high-speed motors. However, this study did not fully consider the effect of relaxation, and we hope we can improve on this in subsequent studies.

## Figures and Tables

**Table 1:** Basic parameters of sample preparation materials.

Property	Nominal Value
Brand	1K101
Supplier	Antai Technology & Materials Co., Ltd.
Nominal Composition	Fe${}_{80}$Si${}_{9}$B${}_{11}$
Width (mm)	60
Thickness (μm)	24 ± 2
Induction B${}_{800}$ (T)	≥1.56
Induction B${}_{80,50\;\mathrm{Hz}}$ (T)	≥1.40
Coercivity (A/m)	≤2.0
Iron Loss P${}_{50\;\mathrm{Hz},1.4T}$ (W/kg)	≤0.12
Curie temperature (°C)	428
Crystallization temperature (°C)	525
Electrical Resistivity (μΩ·m)	1.40

**Table 2:** Measured and calculated loss of samples after W-EDM at different frequencies.

Frequency	Iron Loss Density, P/W·kg${}^{-1}$			Prediction Accuracy
f/Hz	Nominal	Measured	Estimated	Improvement
50	0.1886	0.2306	0.2730	1.03%
100	0.3520	0.4682	0.4893	81.9%
200	0.8461	1.0073	1.0967	44.6%
400	2.0433	2.4032	2.4424	89.1%

## Data Availability

The data presented in this study are available upon request from the corresponding author.

## Abbreviations

The following abbreviations are used in this manuscript:

W-EDM	Wire-cut electric discharge machining
AA	Amorphous alloy
